# Investigating the effectiveness of iron nanoparticles synthesized by green synthesis method in chemoradiotherapy of colon cancer

**DOI:** 10.1016/j.heliyon.2024.e28343

**Published:** 2024-03-16

**Authors:** Farshad Seyed Nejad, Mostafa Alizade-Harakiyan, Mehdi Haghi, Rokhsareh Ebrahimi, Mohammad Mahdi Zangeneh, Alireza Farajollahi, Roghayeh Fathi, Reza Mohammadi, Samira Samadi Miandoab, Mohammad Heydarnezhad Asl, Parina Asgharian, Baharak Divband, Amin Ahmadi

**Affiliations:** a ‏Biotechnology Research Center, Tabriz University of Medical Sciences, Tabriz, Iran; b Department of Radiation Oncology, Faculty of Medicine, Tabriz University of Medical Sciences, Tabriz, Iran; c Medical Physics Department, Faculty of Medicine, Tabriz University of Medical Sciences, Tabriz, Iran; d Department of Animal Biology, Faculty of Natural Sciences, University of Tabriz, Tabriz, Iran; e Medicinal Chemistry Department, Faculty of Pharmacy, Tabriz University of Medical Sciences, Tabriz, Iran; f Biotechnology and Medicinal Plants Research Center, Ilam University of Medical Sciences, Ilam, Iran; g Medical Radiation Science Research Team, Tabriz University of Medical Sciences, Tabriz, Iran; h Polymer Research Laboratory, Department of Organic and Biochemistry, University of Tabriz, Tabriz, Iran; i Department of Pharmacognosy, Faculty of Pharmacy, Tabriz University of Medical Sciences, Tabriz, Iran; j Department of Inorganic Chemistry, Faculty of Chemistry, University of Tabriz, Tabriz, Iran; k Research Center for Pharmaceutical Nanotechnology, Biomedicine Institute, Tabriz University of Medical Sciences, Tabriz, Iran

**Keywords:** Iron nanoparticles, *Mentha spicata* extract, Colon carcinoma, Radiotherapy

## Abstract

Current methods of colon cancer treatment, especially chemotherapy, require new treatment methods due to adverse side effects. One important area of interest in recent years is the use of nanoparticles as drug delivery vehicles since several studies have revealed that they can improve the target specificity of the treatment thus lowering the dosage of the drugs while preserving the effectiveness of the treatment thus reducing the side effects. The use of traditional medicine has also been a favorite topic of interest in recent years in medical research, especially cancer research. In this research work, the green synthesis of Fe nanoparticles was carried out using Mentha spicata extract and the synthesized nanoparticles were identified using FT-IR, XRD, FE-SEM and EDS techniques. Then the effect of *Mentha spicata*, Fe nanoparticles, and *Mentha spicata* -loaded Fe nanoparticles on LS174t colon cancer cells, and our result concluded that all three, especially *Mentha spicata* -loaded Fe nanoparticles, have great cytotoxic effects against LS174t cells, and exposure to radiotherapy just further intensified these results. The in vitro condition revealed alterations in the expression of pro-apoptotic BAX and anti-apoptotic Bcl2, suggesting a pro-apoptotic effect from all three components, particularly the *Mentha spicata-loaded* Fe nanoparticles. After further clinical trials, these nanoparticles can be used to treat colon cancer.

## 1 Introduction

**Scheme 1:**
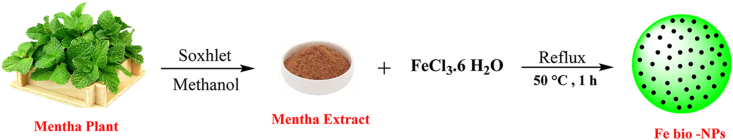
Preparation of Fe bio nano particles (Fe bio-NPs).

Colon cancer is one of the top five most common cancer both in incidence and mortality rates. This cancer is 30%–40% more relevant in men than women and it is also mostly relevant in modern world countries [[1], [2], [3]]. High-fat diets, non-active lifestyles, obesity, and tobacco consumption are some of the risk factors associated with colon cancer [4]. Chemotherapy is the most common treatment method used to treat this cancer but chemotherapy agents usually have severe side effects that tantalize patients even after the treatment is over. These side effects include but are not limited to fatigue, nausea, and immune system weakness [5]. It's because of these side effects that researchers have been looking for alternative treatment methods and herbal medicine is an interesting area. The use of natural recourses eliminated the necessity of toxic chemicals for the large-scale preparation of advanced metal nanoparticles due to the presence of polyphenols, reducing sugars, amino acids and other antioxidants in their extract, which prevent unwanted growth [6]. Plants extract to contains alkaloids, amino acids, enzymes, flavonoids, polyphenols, proteins, reducing sugars and other bioactive components could be involved in metal ions reduction, formation and stabilization of metal NPs in an aqueous solution [7]. Plants extract are less toxic and require minimum purification when compared to chemical methods [8]. Biologically synthesized nanoparticles showed great promise for cancer therapy, higher antimicrobial activities, biological labeling, catalysis, dye sensitized solar cells and detection of tumor [9]. *Mentha spicata* (MS) is a member of the Lamiaceae family and is known for secreting secondary metabolites that are responsible for its anti-microbial, anti-oxidant, anti-inflammatory, and anti-cancer effects [10]. One other area that is important in lowering the side effects of cancer treatment is efficiency and specificity. Having our therapeutic compounds target the interest area efficiently and specifically will reduce the amount of therapeutic agent needed which will lower side effects and financial costs. Nanoparticles (NPs) like Iron nanoparticles (FeNPs) have been showing some promising results in this area [11]. In the case of FeNPs, their easy preparation, facile chemical functionalization, biocompatibility, and low toxicity make them great carriers for drug delivery, especially in cancer therapy [11,12]. On the other hand, although radiotherapy is not a recommended method of treatment for colon cancer its effectiveness with other cancers and its combination with novel methods may be an asset to new and alternative treatments [13] (see Scheme 1).

In recent years, advances in nanotechnology (especially nanoparticles) have played an important role in radiation sensitizers, and the study of radiation sensitizers has become an important topic in oncology radiotherapy. The entry of nanoparticles into tumor cells increases the local energy given to the tumor, thereby increasing damage to malignant cells and reducing unwanted damage to normal tissue [14,15]. Since materials with high atomic numbers increase the destructive effect of radiation, to increase the radiation response, metal-based nanoparticles have been widely evaluated as a radiation sensitizer for cancer treatment [16]. The interaction of radiation with nanoparticles that have a higher density and atomic number increases the damage caused by radiation by producing secondary electrons such as Compton, photoelectron, and Auger electrons due to their higher scattering and absorption cross-sections [17]. For this purpose, scientists propose materials with high atomic numbers on the nanoscale to increase the absorption dose. In recent years, the effects of plant-based nanoparticles have been used in the treatment of various cancers [18,19]. Due to their very small size, nanoparticles can be easily distributed through the bloodstream and thus have a homogeneous distribution throughout the tumor. As a result, nanoparticles are used as radiation sensitizers in studies. There have been very few studies on the effect of nanoparticles on radiation therapy in the past, and there is no study on the effect of herbal nanoparticles on radiation therapy [20].

This study aims to investigate the effects of MS and FeNPs, both alone and combined, on colon cancer cell line ls174t with and without radiation to determine the effectiveness of natural medicine combined with NPs with and without radiation to conclude if this new approach to cancer has the potential to open new horizons in cancer therapy.

## 2 Experimental

### 2.1 Preparation of Fe bio nanoparticles (Fe bio-NPs)

In this section, deionized water was used for the green synthesis of Fe NPs. 10 ml of FeCl_3_. 6H_2_O at a concentration of 0.05 M was combined with 40 ml of an aqueous extract solution (100 μg/ml). For 1 h, the mixture was refluxed at 50 °C. Iron nanoparticle production was shown by the hue changing from yellow to black. The precipitate was triplet rinsed in water and then centrifuged for 15 min at 10,000 rpm. For chemical analysis and assessment of its biological activity, the resulting black powder was maintained in a vial.

### 2.2 Cell viability assay

Ls174t colon cancer cells were purchased from the Pasteur Institute of Iran. These cells were grown in RPMI1640 media supplemented with fetal bovine serum (FBS), l-glutamine, a combination of antibiotics (streptomycin and penicillin), and non-essential amino acids. The culture was then kept at 37 °C in a CO_2_-filled environment.

The methyl thiazole tetrazolium (MTT) assay kit purchased from arsamsysbio.com, Iran was used to assess cell viability. After transferring the cells to 96-well plates and treating them with 500, 1000, 2000, and 4000 μg/ml of MS extract, FeNPs, and MS-loaded FeNPs for 24, 48, and 72 h, the assay was performed according to the instructions.

### 2.3 mRNA extraction and expression assay

mRNA extraction was done using ZiAzole (ZiAViZ company, Iran) mRNA extraction kit and according to their instructions. Next, cDNA was produced by RT-PCR utilizing the cDNA Synthesis Kit (Yekta Tajhiz, Iran).

The qPCR kit from Yekta Tajhiz was purchased as well for the expression assay. 0.5, 1, 2, and 4 μg/ml of MS extract, FeNPs, and MS-loaded FeNPs were administered to Ls174t. The expression of BAX and Bcl_2_ apoptotic genes were analyzed and beta-actin was measured as a reference gene. Primer sequences used for this assay can be seen in Table 1.

**Table 1:** Primer sequences used in qPCR.

Gene	Forward	Reverse
Bata actin	5′- GGAGTCCTGTGGCATCCACG- 3′	5’ – CTAGAAGCATTTGCGGTGGA – 3′
BAX	5′- GGCCCACCAGCTCTGAGCAGA- 3′	5′– GCCACGTGGGCGGTCCCAAAGT – 3′
Bcl-2	5′- GTGGAGGAGCTCTTCAGGGA-3′	5′- AGGCACCCAGGGTGATGCAA-3′

### 2.4 Radiotherapy

**Fig. 1:**
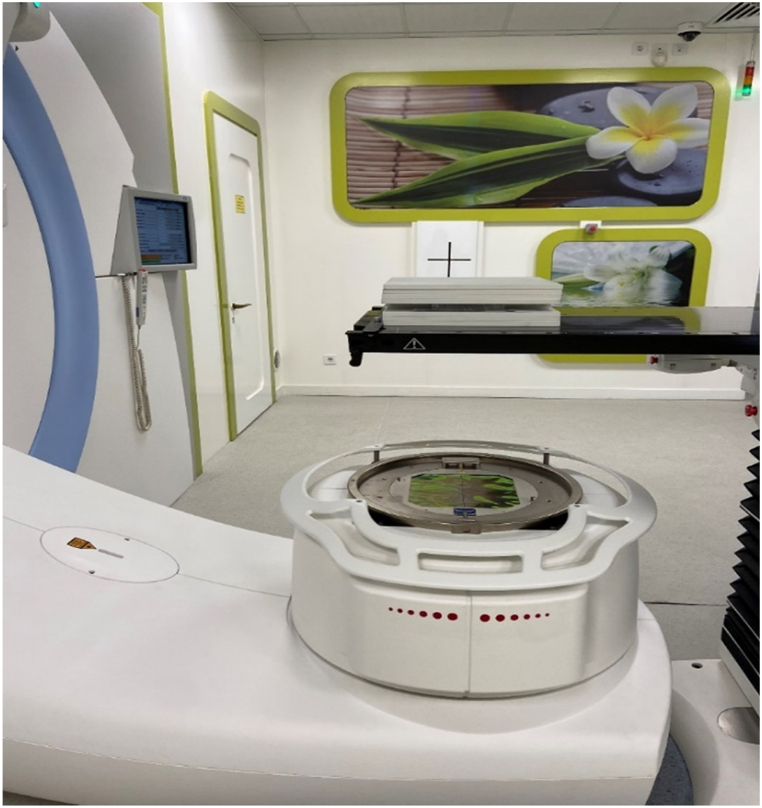
Image of radiotherapy machine.

High-energy X-ray irradiation of cells at 6 and 15 MV energies was performed by the linear accelerator of the elekta synergy platform (manufactured by Sweden) in the radiooncology department of Shahid Madani Hospital in Tabriz, with dose of 2Gy in a SSD of 100 cm. It is worth mentioning that to create the conditions of sufficient build-up and adequate back scatter of the beam, polystyrene was used to be placed under and on the plate (Fig. 1).

## 3 Results and discussion

### 3.1 Characterization of synthesized materials

#### 3.1.1 FT-IR study

**Fig. 2:**
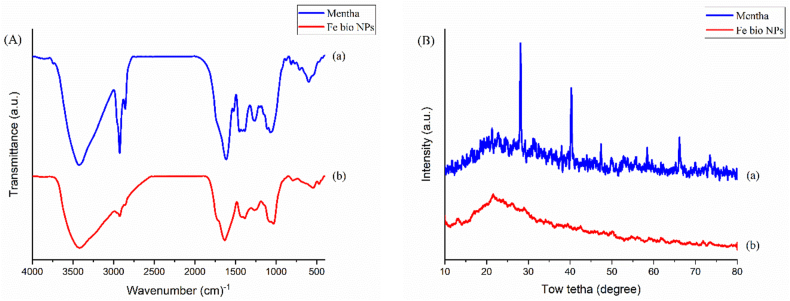
(A) FT-IR spectra of Mentha extract (a), Fe bio-NP_S_ (b) and (B) XRD spectra of Mentha extract (a), Fe bio-NP_S_ (b).

The Mentha extract and Fe bio-NPS FT-IR spectra were graphed, and this allowed for the identification of potential biomolecules in the plant extract that could be the cause of the decreasing Fe ions and act as capping agents. Fig. 2 (A) shows the Mentha's FTIR graph before and after the Fe NPs were synthesized. See the spectra at 1070, 1263, 1384, 1451, 1614, 2857, 2925, and 3422 cm^−1^ for the Mentha extract absorption bands. It is probable for linking the aliphatic amines' C–N stretching vibration to the band at 1070 cm^−1^. The band at 1451 cm^−1^ is caused by a C=O stretching vibration, which can come from the functional groups of ketones, aldehydes, and carboxylic acids. The C=C stretching frequencies of the aromatic ring, which is a component of phenolic compounds (such as flavonoids and polyphenols), are responsible for the bands seen at 1614 cm^−1^. At 2925 cm^−1^, the absorption band linked to the stretch and C–H vibrations of aliphatic hydrocarbon chains is visible. Strong hydrogen bonding is indicated by the wide bands at 3422 cm^−1^, which are the result of the phenolic compounds' O–H stretching. These functional groups demonstrate the existence of organic acids, phenols, and aliphatic amines in the extracts, which may serve as stabilizing and reducing agents during the synthesis of Fe bio NPs. The bands for functionalized Fe-bio NPs (Fig. 2A (b)) are identical to those for Mentha extract (Fig. 2A (a)) with a small shift, according to the FTIR spectra. The IR spectra shows that the molecular bonds of the functional groups in the extract components have not changed significantly. When Fe_3_O_4_ and Fe_2_O_3_ dissolve, little amounts of Fe–O are produced, which is why the FTIR spectra of Fe-bio NPs shows an absorption band at about 470 cm^−1^. This could be connected to the production of Fe-bio NPs that have been exposed to water or air and have partially oxidized [6]. In bulk magnetite, the Fe–O stretching band usually appears at 544 cm^−1^. Because of the finite size of nanoparticle synthesis, the band shifts to higher wavenumbers (582.82 cm^−1^) [11,12].

#### 3.1.2 XRD study

X-ray diffraction (XRD) technique was used to characterize the Fe-Bio-NPS formation (Fig. 2B). The diffraction peaks at 2θ of 28.1°, 40.28°, 47.4°, and 66.16° observed in the crude Mentha extract's XRD diffractogram may be related to its semi-crystalline structure (Fig. 2B(a)) [4]. The XRD pattern of the produced Fe-bio NPs, as illustrated in Fig. 2B(b), reveals the absence of any diffraction peaks, even at the distinctive zero-valent iron peak, 2θ value of 44.9°. This conclusion is in line with the findings of other investigations and validates the amorphous character of the green produced Fe-bio NPs. The presence of many biomolecules in the plant extract that function as capping and reducing agents could be the reason for the emergence of a broad band at a 2θ value of 44.9° [12].

##### 3.1.2.1 SEM study

**Fig. 3:**
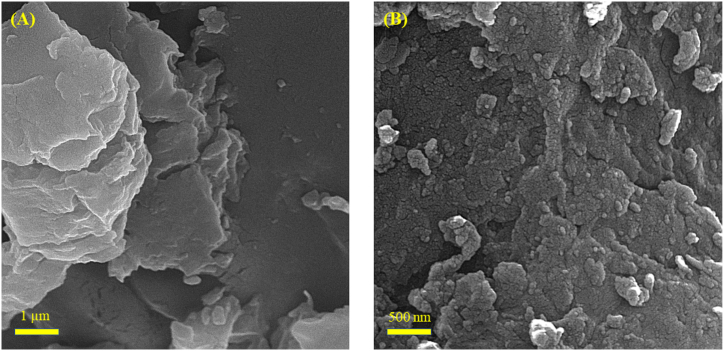
SEM micrographs of Mentha extract (A), Fe bio-NP_S_ (B).

Using the SEM method, the surface morphology of Fe-bio-NPs and Mentha extract was described (Fig. 3). The surface shape of the Mentha extract exhibits an almost smooth surface, as seen in Fig. 3A. On the other hand, the morphology of Fe-bio-NPs exhibits coarse structures, suggesting that Fe-NPs is present on the plant extracts' surface (Fig. 3B).

##### 3.1.2.2 EDX analysis

**Fig. 4:**
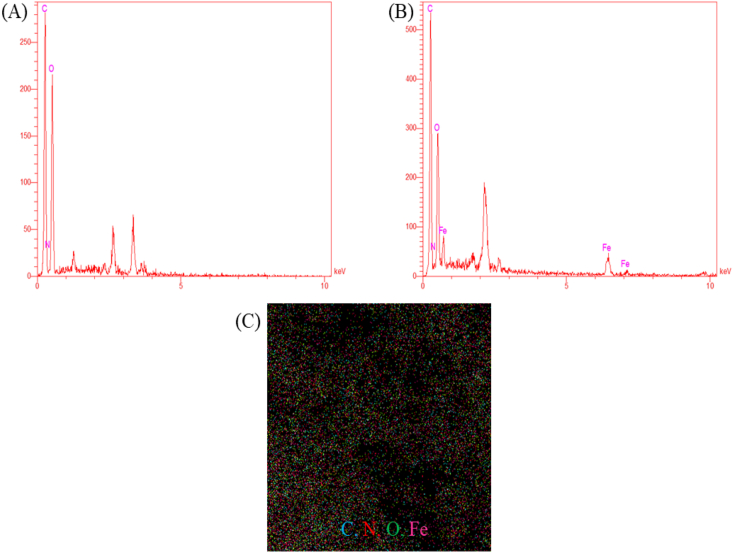
EDX spectra of Mentha extract (A), Fe bio-NP_S_ (B) and Mapping image of Fe bio-NP_S_ (C).

Using the EDX method, the elemental analysis of produced substances was investigated (Fig. 4). The successful creation of these nanoparticles was confirmed by the presence of the Fe element in the EDX spectrum of Fe-bio NPs when compared to Mentha extract (Fig. 4A) and (B)). Concurrently, the mapping image of Fe-bio NPs verifies the presence of various percentages of iron, carbon, oxygen, and nitrogen atoms (Fig. 4C).

### 3.2 Cell viability

As mentioned above, the MTT assay was carried out to assess cell viability after treatment with various methods. IC50 values are present in Table 2.

**Table 2:** The IC50 results for the MTT assay.

	MS	Nanoparticle	Nanoparticle + MS
	24 h of treatment (μg/ml)		
Without radiation	4165	4050	2740
With 2Gy and 6 MV of radiation	2505	2892	1896
With 2Gy and 15 MV of radiation	2348	2319	1085
	48 h of treatment (μg/ml)		
Without radiation	3465	3301	2299
With 2Gy and 6 MV of radiation	2387	1911	1681
With 2Gy and 15 MV of radiation	2076	2136	1060
	72 h of treatment (μg/ml)		
Without radiation	2205	2101	1589
With 2Gy and 6 MV of radiation	1915	1781	1163
With 2Gy and 15 MV of radiation	1199	1286	574.5

**Fig. 5:**
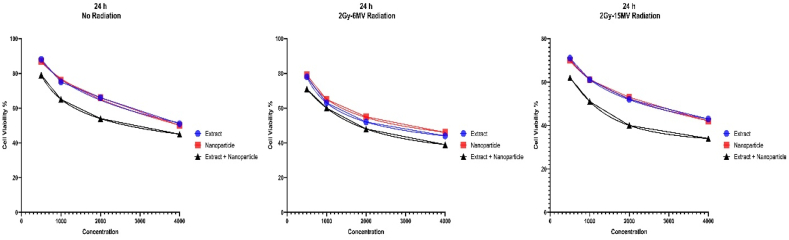
The MTT graphs showing cell viability after 24 h of treatment with various methods. Our cells were also exposed 2Gy and 6 MV and 2Gy and 15 MV of radiation compared to no radiation. To All components were cytotoxic to cancer cells as demonstrated but MS-loaded FeNPs had the most cytotoxic effect by far.

**Fig. 6:**
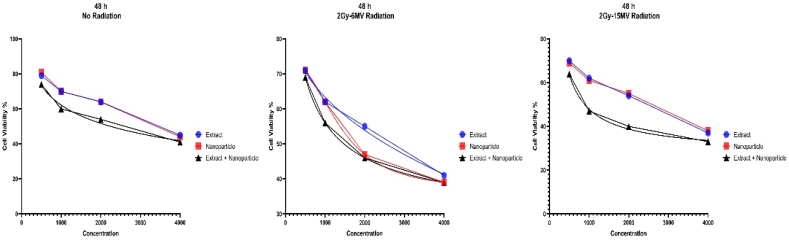
The MTT graphs showing cell viability after 48 h of treatment with various methods. Our cells were also exposed 2Gy and 6 MV and 2Gy and 15 MV of radiation compared to no radiation. All components were cytotoxic to cancer cells as demonstrated but MS-loaded FeNPs had the most cytotoxic effect by far.

**Fig. 7:**
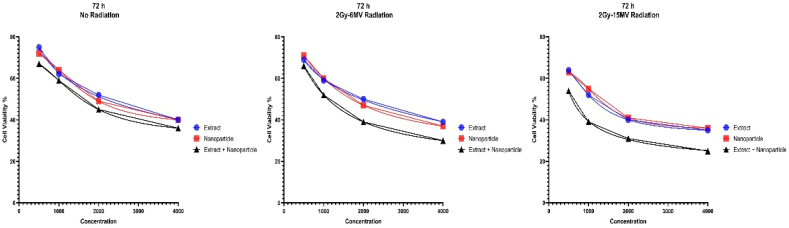
The MTT graphs showing cell viability after 72 h of treatment with various methods. Our cells were also exposed 2Gy and 6 MV and 2Gy and 15 MV of radiation compared to no radiation. All components were cytotoxic to cancer cells as demonstrated but MS-loaded FeNPs had the most cytotoxic effect by far.

The graphs showing the decrease in cell viability due to each method of treatment are also presented below (Fig. 5, Fig. 6, Fig. 7):

Considering the information presented in the graphs and the IC50 values, it's obvious that all our treatment agents have cytotoxic effects on our cells, especially MS-loaded FeNPs. Furthermore, high doses in longer periods present more toxic results against cancer cells. Even more, it seems that radiotherapy helps this process immensely and can be a component of this new treatment method.

### 3.3 Expression assay

**Fig. 8:**
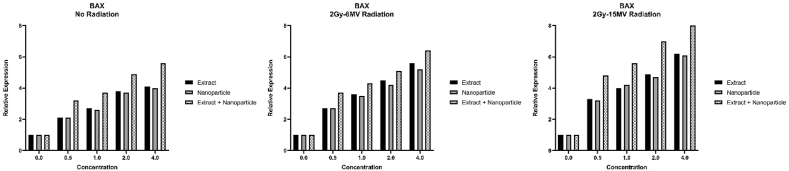
Expression of BAX after various treatments (p-value<0.05: no radiation: **, 2Gy-6MV: ***, 2Gy-15MV: ***). Combined effect of radiation and MS-loaded FeNPs can greatly increase the expression of BAX pro apoptotic gene.

**Fig. 9:**
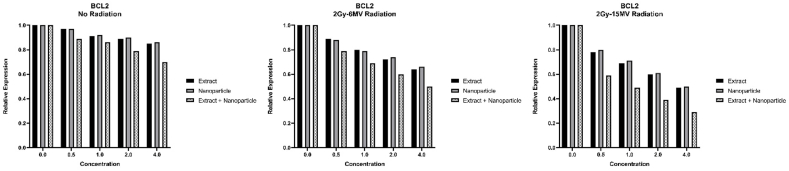
Expression of Bcl_2_ after various treatments (p-value<0.05: no radiation: *, 2Gy-6MV: **, 2Gy-15MV: ***). Combined effect of radiation and MS-loaded FeNPs can greatly decrease the expression of Bcl2 anti apoptotic gene.

Expression of BAX and Bcl_2_ was measured after various methods of treatment and the graphs showing the results are presented below (Fig. 8, Fig. 9).

Looking at the graphs above and p-values obtained from analyzing the results, we can conclude that all three components, especially MS-loaded FeNPs, have great anti-cancer capabilities, especially in higher doses since the expression of pro-apoptotic BAX has increased and the expression of anti-apoptotic Bcl_2_ has decreased after treatment. We can also see that radiotherapy has positively impacted the performance of our components and has increased the anti-cancer properties of these components.

This study aimed to investigate the anti-cancer potential of MS-loaded FeNPs against LS174t colon cancer cells in the hope of finding an alternative treatment route for this disease. We treated our cells with MS, FeNPs, and MS-loaded FeNPs. Our results showed that all three components had acceptable cytotoxic effects against these cells especially MS-loaded FeNPs. We also exposed our cells to different intensities of radiotherapy and it too had negative effects on cell viability.

One study conducted by Yasushi Nakamura et al. used the *n*-hexane extract of *Mentha spicata* leaves containing piperitenone oxide, which is responsible for MS odder, against the RCM-1 human colon cancer cell line. This study concluded that piperitenone oxide was successfully able to induce differentiation in these cells [21]. Another 2016 study treated HepG2, Caco2, and MCF-7 cancer cells with different herbal extracts including MS, and concluded that it has anti-proliferative effects on all three cancer cell lines [22]. Sanaa K. Bardaweel et al. also treated T47D, HCT-116, and MCF-7 with MS essential oil and reported great anti-proliferative and anti-oxidant activity [23]. MS extract was reportedly very cytotoxic against A-549, COLO-205, HCT-116, MCF-7, NCI–H322, PC-3, THP-1 and U-87MG cancer cell lines in a 2014 study. This extract presented 70–97 % cytotoxicity against these cell lines [24]. Combination of the results obtained from our study and the studies presented above along with the fact that NPs have been proven to improve the specificity of drug delivery and enhance its effectiveness we can safely assume that all three components, especially MS-loaded FeNPs, are great candidates for colon cancer therapy, especially with the help of radiotherapy.

We also report an increase in the expression of BAX and a decrease in the expression of Bcl_2_ as a result of treatment with all three components, especially MS-loaded FeNPs. The upregulation of BAX has been reported before [25] but sadly not many studies have investigated the anti-apoptotic effects of MS extract through the regulation of BAX and Bcl_2_.

## 4 Conclusion

This study aims to investigate the effects of MS and FeNPs, both alone and combined, on colon cancer cell line ls174t with and without radiation. When Fe_3_O_4_ and Fe_2_O_3_ dissolve, little amounts of Fe–O are produced, which is why the FTIR spectra of Fe-bio NPs shows an absorption band at about 470 cm^−1^. This could be connected to the production of Fe-bio NPs that have been exposed to water or air and have partially oxidized. In bulk magnetite, the Fe–O stretching band usually appears at 544 cm^−1^. Because of the finite size of nanoparticle synthesis, the band shifts to higher wavenumbers (582.82 cm^−1^). Diffraction peaks at 2θ of 28.1°, 40.28°, 47.4°, and 66.16° were visible in the crude Mentha extract's XRD diffractogram, and these could be indicative of its crystal structure.

In the in vitro condition, the expression of pro-apoptotic BAX and anti-apoptotic Bcl_2_ was also changed which indicates a pro-apoptotic activity from all three components, especially MS-loaded FeNPs. Further and more in-depth studies can shed more light on this subject and hopefully help patients for a better recovery.

## Funding

This research received no external funding.

## Ethics approval and consent to participate

The ethical approvals of this study have been considered based on the criteria of Heliyon Journal. Also, this study was approved by the Ethical Committee of Tabriz University of Medical Sciences (IR.TBZMED.VCR.REC.1400.244) and (No.66942).

## Data Availability statement

Data included in article/supp. material/referenced in article.

## CRediT authorship contribution statement

**Farshad Seyed Nejad:** Methodology, Investigation. **Mostafa Alizade-Harakiyan:** Writing – review & editing, Writing – original draft, Supervision, Methodology. **Mehdi Haghi:** Investigation, Formal analysis. **Rokhsareh Ebrahimi:** Formal analysis, Data curation. **Mohammad Mahdi Zangeneh:** Conceptualization. **Alireza Farajollahi:** Validation, Supervision. **Roghayeh Fathi:** Resources, Methodology, Conceptualization. **Reza Mohammadi:** Conceptualization. **Samira Samadi Miandoab:** Project administration. **Mohammad Heydarnezhad Asl:** Writing – original draft, Methodology. **Parina Asgharian:** Data curation. **Baharak Divband:** Formal analysis. **Amin Ahmadi:** Investigation.

## Declaration of competing interest

The authors declare that they have no known competing financial interests or personal relationships that could have appeared to influence the work reported in this paper.
